# Childhood adversity and approach/avoidance-related behaviour in boys

**DOI:** 10.1007/s00702-022-02481-w

**Published:** 2022-03-11

**Authors:** Nicola Grossheinrich, Julia Schaeffer, Christine Firk, Thomas Eggermann, Lynn Huestegge, Kerstin Konrad

**Affiliations:** 1 Child Neuropsychology Section, Department of Child and Adolescent Psychiatry, Psychosomatics and PsychotherapyFaculty of Medicine, RWTH Aachen University, Aachen, Germany; 2 Department of Social Sciences, Catholic University of Applied Sciences of North Rhine‐Westphalia, Aachen, Cologne, Germany; 3 Institute of Psychology, RWTH Aachen University, Jägerstraße 17-19, 52066 Aachen, Germany; 4 Institute of Human Genetics, RWTH Aachen University, Pauwelsstraße 30, 52074 Aachen, Germany; 5 Institute of Psychology, University of Würzburg, Röntgenring 11, 97070 Würzburg, Germany; 6 JARA-Brain Institute II, Molecular Neuroscience and Neuroimaging, Research Center Juelich, Juelich, Germany

**Keywords:** Emotion regulation, Attentional bias, MAOA, Eye tracking

## Abstract

Childhood adversity has been suggested to affect the vulnerability for developmental psychopathology, including both externalizing and internalizing symptoms. This study examines spontaneous attention biases for negative and positive emotional facial expressions as potential intermediate phenotypes. In detail, typically developing boys (6–13 years) underwent an eye-tracking paradigm displaying happy, angry, sad and fearful faces. An approach bias towards positive emotional facial expressions with increasing childhood adversity levels was found. In addition, an attention bias away from negative facial expressions was observed with increasing childhood adversity levels, especially for sad facial expressions. The results might be interpreted in terms of emotional regulation strategies in boys at risk for reactive aggression and depressive behaviour.

## Introduction

Over the past decades, conduct and emotional problems have increased in youth of the western industrialized world (Collishaw et al. 2004) and have been associated with severe problems at school, difficulties with integration into work life, chronic health problems, substance abuse and delinquency. Thus, the associated costs for society are tremendous (Bonin et al. 2011) indicating the need to investigate the underlying neurocognitive mechanisms that facilitate the emergence of emotional problem behaviour in children.

One model of aggressive behaviour can be derived from the social information processing theory by Crick and Dodge (1994). According to the model, aggressive children are assumed to interpret ambiguous information in a more hostile manner and to attend more frequently to hostile cues (Crick and Dodge 1996). Whereas the hostile attribution bias has been investigated extensively (Orobio de Castro et al. 2002), the hostile attention bias has been observed in a few studies investigating undergraduates and violent offenders who scored high on trait anger (Honk et al. 2001a, b; Smith and Waterman 2003, 2004). Interestingly, in experimental designs a hostile attention bias has been detected in adults only when anger was induced (Eckhardt and Cohen 1997; Cohen et al. 1998), which is early evidence that the so-called hostile attention bias could not only be a precursor but also a consequence of anger.

In a similar vein, a negative bias of information processing is assumed to cause negative mood and thoughts eventually leading to depression (Fritzsche et al. 2010). The concept of a negative bias in social cognition for the development of major depressive disorder (MDD) was postulated in the theoretical framework of Beck’s schema theory of depression (e.g. Beck 1967, 1976), which initiated a controversial debate. In detail, clinicians and researchers assume that the negative information bias represents a stable trait, which precedes sad mood (Fritzsche et al. 2010). In contrast, others refer to the negative bias as transient that co-occurs with sad mood (Lewinsohn et al. 1981) and fades away when sad mood is upregulated. The latter argument was proven by experimental settings in healthy adults (Isaacowitz et al. 2008) and in children (Grossheinrich et al. 2018).

Causes and effects of social cognition processes are in general confounded in clinical groups. Hence, it follows that the investigation of risk factors for mental disorders seems to be more suitable to understand the underlying underpinnings of aberrant emotional problem behaviour.

One of the most widely studied risks for reactive aggression and depressive behaviour is childhood adversity, which is supposed to alter underlying neurocognitive mechanisms through chronic exposure to stress (Lupien et al. 2009). It is well documented that chronic stress exposure leads to enhanced stress sensitivity (McCrory et al. 2010) and a decreased positive affect (Stellern et al. 2014) accompanied by aberrant neural activations for the avoidance (Maheu et al. 2010) and the approach (reward) system (Dillon et al. 2009; Mehta et al. 2010).

With respect to aggressive behaviour, the effect of childhood adversity is moderated by the monoamine oxidase (MAOA) genotype, as shown in a path-breaking longitudinal study by Caspi et al. (2002). While the reported MAOA childhood adversity × genotype interaction has been successfully replicated in several studies, other researchers have failed to reproduce this result. A meta-analysis shed light on the contradictory findings and confirmed that childhood adversity predicted antisocial outcomes more strongly for the low-activity genotype (MAOA-L) relative to the high- activity genotype (MAOA-H), especially for male carriers (Byrd and Manuck 2014), while the underlying neurocognitive underpinnings remained unsolved.

Neurofunctional imaging studies suggest that childhood adversity and the MAOA-L genotype increase the risk of altered stress sensitivity (Maheu et al. 2010), as evidenced by heightened amygdala reactivity, which is assumed to trigger reactive aggressive behaviour (Meyer-Lindenberg et al. 2006) and which is discussed as a potential precursor for a depressive development (Swartz et al. 2015). Moreover, increasing amygdala reactivity has been replicated in male MAOA-L carriers with increasing childhood adversity levels (but not in female MAOA-L carriers; Holz et al. 2016).

Due to small effect sizes, behavioural studies investigating genetics and altered stress sensitivity usually involve large samples (Byrd and Manuck 2014; Kim-Cohen et al. 2006; Karg et al. 2011). In contrast, the effect size of a meta-analysis investigating the attention bias for emotional information in dependence of the serotonin transporter (5-HTTLPR) was estimated to be moderate (Pergamin-Hight et al. 2012), suggesting the attention bias could serve as an intermediate phenotype of stress sensitivity.

All things considered, the previous data suggest a potentially important role of childhood adversity in the development of abnormal emotional behaviour such as reactive aggression or depressive symptoms. Childhood adversity might lead to altered stress sensitivity and social information processing styles characterized, e.g., by facilitated processing of threat stimuli (e.g. Gibb et al. 2009; Lakshman et al. 2020; Pollak and Sinha 2002) or by disengagement problems from sad emotions (Romens and Pollak 2012). However, still little is known how childhood adversity (in the low to medium ranges) affects social information processing in typically developing children. For this reason, the contribution of childhood adversity should be further clarified through the study of intermediate phenotypes—such as attention biases—in children before mental disorders emerge. Hence, we examined the influence of childhood adversity on attention biases in boys using an eye-tracking paradigm (Grossheinrich et al. 2018) in which a spontaneous gaze towards different facial emotional expressions was analysed.

In particular, the attention bias to negative stimuli in general is assumed to be associated with stress sensitivity (Fox et al. 2011; MacLeod et al. 2002). In children, an attention bias away from negative stimuli was repeatedly reported (Boyd et al. 2006; Gibb et al. 2011, 2016; Harrison and Gibb 2015; Kujawa et al. 2011), including one study that examined children with a genetic risk for heightened stress sensitivity (Gibb et al. 2011). Thus, we assume that children look away from negative (angry, fearful, sad) social cues with increasing childhood adversity levels and that the MAOA genotype moderates the effect of childhood adversity.

According to the attention bias for positive (happy) facial expressions (which is related to the approach/reward system; Shechner et al. 2012), childhood adversity is associated with decreased positive affect (Stellern et al. 2014). Thus, if children tend to upregulate their mood, they should show an attention bias towards happy facial expressions with increasing childhood adversity levels.

## Methods

### Participants

In total, the data from 61 boys (aged 6 to 13 years, *M* = 9.44; SD 1.48) were analysed. The subjects were recruited in order to enhance chance to get a wide range of childhood adversity levels, schools from varying neighbourhoods were addressed. Subjects were asked to be free of any current mental disorder. Only participants with normal vision and an IQ score > 70 were included. Participants whose eye movements could not be tracked successfully for at least 75% of the trials were excluded (*N* = 2, initial sample: *N* = 63; Isaacowitz et al. 2008). Boys were divided into two groups carrying the 3-repeat-alleles (MAOA-L, *N* = 25) or the 4-repeat-alleles (MAOA-H, *N* = 36) polymorphisms.

Raven’s Coloured Progressive Matrices test (CPM, Raven 1947; translated by Bulheller and Häcker (2002)) was administered to screen the participants’ intelligence. The children’s temperaments were assessed by the temperament scales of the Junior Temperament and Character Inventory (JTCI 7-11R, Luby et al. 1999; German version: Goth and Schmeck 2009) according to the biosocial model postulated by Cloninger (1986). Behavioural problems were evaluated using the Child Behaviour Checklist (CBCL/4–18, Achenbach 1991; Arbeitsgruppe Deutsche Child Behaviour Checklist 1998). The two genotype groups did not differ with respect to age, intelligence and most of the temperament scores. However, children carrying the MAOA-H polymorphism exhibited higher scores on scales indicating externalizing behaviour problems (CBCL) and internalizing behaviour problems (CBCL, JTCI ‘harm avoidance’), and displayed higher childhood adversity levels.

The study was conducted in accordance with the Declaration of Helsinki and was approved by the local Ethics Committee. The parents provided written informed consent and children’s assent was obtained.

### Materials and procedure

#### Childhood adversity

Chronic adverse events in childhood are supposed to alter underlying neurocognitive mechanisms through chronic exposure to stress hormones (Lupien et al. 2009) leading to enhanced stress sensitivity (McCrory et al. 2010) and a decreased positive affect (Stellern et al. 2014). As retrospective judgements of early childhood experiences might be viewed critically (Reuben et al. 2016), children were investigated bearing a high probability of parents’ socially desirable responses. In order to avoid parents’ socially desirable responses and to explore childhood adversity in the general population, the German version (Eltern-Belastungs-Screening zur Kindeswohlgefährdung, EBSK, Deegener et al. 2009) of the child abuse potential inventory (CAPI, Milner 1986) was applied. The CAPI is an instrument—widely used and empirically validated—to detect potential physical child abuse in a variety of situations with demonstrated long-term stability (Milner 1994). The instrument relates to parental risk factors for child maltreatment such as unhappiness, rigid education style, perception of the child as problematic, parental life frustration and dissatisfaction with interpersonal relationships, disruptive family life and a lack of social support. Psychometric criteria of the German version are satisfactory (Deegener et al. 2009). In contrast to the English version, a factor analysis confirmed a single-factor structure in the German version, interpreted as distress. High internal consistency of the distress scale has been reported (Cronbach’s *α* = 0.91). Here, the standardised total score for the normal/unstressed population was used, which was likewise representative in the current sample (M = 50.93; SD 10.81). The index of parent’s socially desirable response was below the cut-off (*L* > 3) for all subjects.

#### Paradigm

Boys viewed a pair of faces on a screen depicting a neutral facial expression paired with either a sad, happy, angry or a fearful facial expression (Grossheinrich et al. 2018).

In total, eight adult and eight child identities (balanced in terms of gender) for all four emotional facial expressions were presented twice (once on the right and once on the left side). Emotional facial expression (sad, happy, angry, fearful), type of stimuli (8 children, 8 adults) and the position of the emotional facial expression (left, right) resulted in 128 trials (4 × 16 × 2), which were presented in a randomised order. A test run was conducted prior to the experiment.

The face stimuli were displayed for 4000 ms, followed by an animated fixation stimulus that showed a cat running in place, which was presented for 500 ms. After each block consisting of ten trials in which the participant was instructed to look thoroughly at the faces on the screen, the calibration process was carried out. The duration of the experiment was approximately 30 min.

The facial stimuli were taken from the Radboud Faces Database (Langner et al. 2010). To avoid habituation effects, facial stimuli from the Pictures of Facial Affect database by Ekman and Friesen (1976) were applied for the test run.

#### Eye tracking

An EyeLink 1000 eye tracker by SR Research Ltd. (Mississauga, ON, Canada) was utilised to measure the pupil and the corneal reflection of the eye. A chin and forehead rest placed on a tabletop in front of the display monitor was used to attain a stable position of the participants’ heads. Eye movements were recorded monocular with a sampling rate of 500 Hz. Calibrations utilized five calibration points that covered the whole visual field in which the stimuli were presented. Finally, oculomotor parameters were extracted using Data viewer software (SR Research).

#### Genotyping

DNA was extracted from buccal swabs. For MAOA-genotyping, standard polymerase chain reaction (PCR) amplification was carried out in a total volume of 25 µl containing 50 ng genomic DNA, 1 unit recombinant TaqPolymerase (Invitrogen, Darmstadt/Germany), PCR buffer (10 mM Tris–HCL, 50 mM KCL, 2.5 mMMgCl2 pH 8.3), 200 mM dNTPs, and 20 pmol of each primer. The MAOA primer sequences were obtained from Sabol et al. (1998), the forward primer was Fam-labeled. Cycling conditions are available on request. PCR products were run via a denaturing capillary gel electrophoresis APPARATUS. The genotyping was performed as suggested by Sabol et al. (1998), who identified a common functional variable number of tandem repeats (VNTR) polymorphism in the promoter region of the MAOA gene (chromosome Xp11.23), leading to the functional classification of the two most common alleles, that is the 3-repeat as low activity (MAOA-L) and the 4-repeat as high activity (MAOA-H).

### Statistical analysis

Equally sized ovals were created around the standardized faces as regions of interest (ROI) and the total fixation duration was analysed. Total fixation duration (fd) was defined as the total time in milliseconds that a subject spent on the ROI of the emotional or neutral facial expression. A ratio score was calculated as

$${\text{Ratio}}\;{\text{score}} = {\text{fd }}\left( {{\text{emotional }}{-}{\text{neutral}}} \right)/{\text{ fd }}\left( {{\text{emotional}} + {\text{neutral}}} \right),$$

which indicated the viewer’s preference for either the emotional or the neutral facial expression during one trial (Isaacowitz et al. 2008). A positive score indicated a preference for the emotional facial expression whereas a negative score points to a preference for the neutral expression. Trials without any fixations on the faces (e.g., due to blinks or temporary signal loss) were excluded from further analysis (*N* = 2).

First, a principal component analysis (PCA) was conducted to reduce data complexity—in particular the four ratio scores referring to four different emotional expressions—to avoid multiple testing. As an underlying rationale, we argued that all negative facial expressions provoke distress as evidenced by heightened amygdala reactivity (Dannlowski et al. 2012, 2013). Then, hierarchical regression models were applied to study the effects of childhood adversity, the MAOA genotype and the childhood adversity x MAOA interaction on each of the two independent factor scores (representing the negative and the positive valence). Post-hoc Pearson’s correlations between each negative emotional facial expression ratio score and the estimated childhood adversity level were calculated.

## Results

### Attention biases to negative and positive facial expressions

**Fig. 1:**
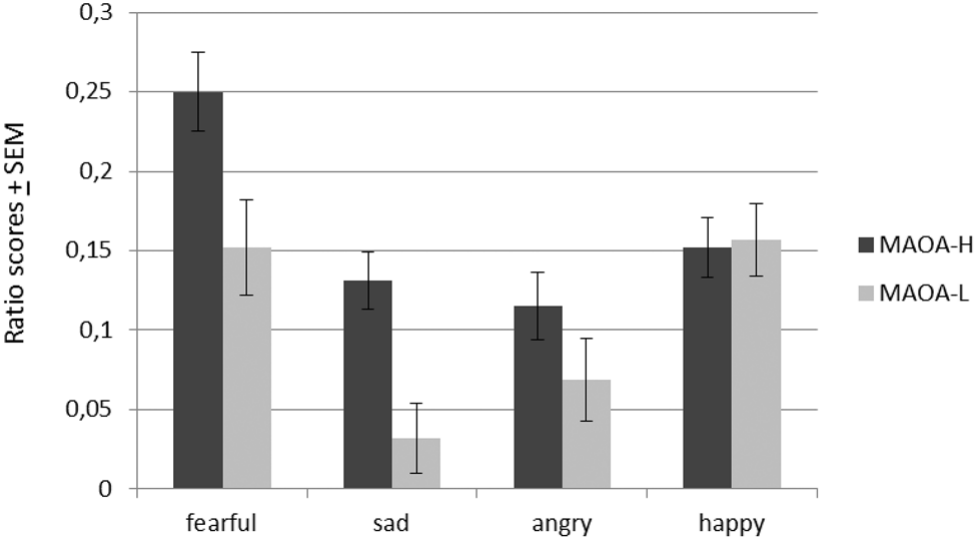
Descriptive statistics (ratio scores) for MAOA-H and MAOA-L carriers. More positive ratio scores indicate greater attentional bias towards emotional faces

Descriptive data of the ratio scores are shown in Fig. 1. To avoid multiple testing, PCA was conducted, which yielded two components (eigenvalue > 1) representing the attention bias to negative facial expressions and positive facial expressions, respectively (see supporting information). The two corresponding factor scores (negative valence, positive valence) served as outcome for two independent hierarchical stepwise regression analyses.

**Fig. 2:**
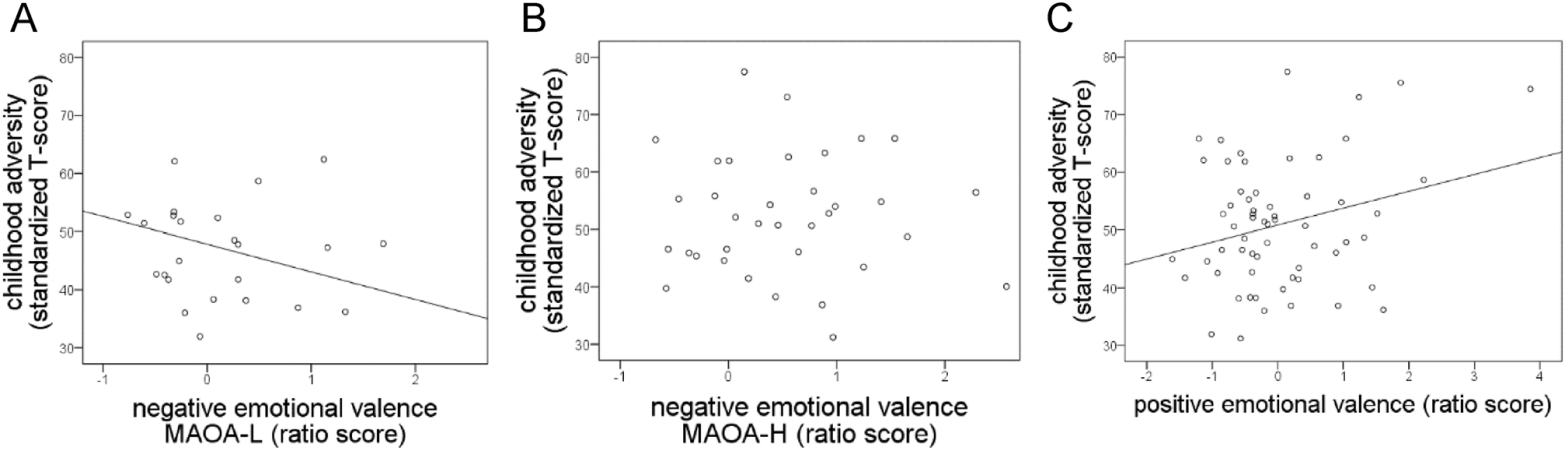
Scatterplots for the relationship between childhood adversity (standardized *T* values) and the negative (**A**, **B**) and positive (**C**) emotional valence (ratio scores). For the negative emotional valence the relationship is illustrated for the MAOA-L (**A**) carriers, while no association could be observed for the MAOA-H genotype

For the negative emotional valence, the childhood adversity × MAOA interaction (*p* < 0.01, adjusted *R*^2^: 16%) and the MAOA genotype (*p* = 0.01, adjusted *R*^2^: 7%) were significant predictors and explained in total 23% of the variance (adjusted *R*^2^). When excluding one outlier (which was three standard deviations apart from the mean), the interaction (childhood adversity × MAOA-genotype) remained, which accounted for 8% (adjusted *R*^2^) of the total variance, while the MAOA genotype (without any interaction) did not survive significance (Fig. 2A). The significant genotype (MAOA) × environment (childhood adversity) interaction emerged from an association between the attention bias ratio score for the negative emotional component and childhood adversity of MAOA-L carriers (*r* = −0.6; *p* < 0.01), while no relation was found for MAOA-H carriers (*r* = 0.01; *p* = 0.58; Fig. 2B).

For the positive emotional component, only childhood adversity was a significant predictor in the model and explained 8% of the variance (adjusted *R*^2^, Fig. 2C), indicating a general preference for positive relative to neutral facial expressions (ratio score) with increasing childhood adversity levels (*r* = 0.31; *p* = 0.02). When age, externalizing or internalizing behaviour were included into the analyses, the overall pattern of results remained unchanged.

For boys carrying the MAOA-L polymorphism, post-hoc Pearson’s correlations (two-tailed) for each negative facial expression yielded a significant negative association between childhood adversity and the total fixation duration for sad faces (*r* = − 0.64, *p* < 0.01). Furthermore, a trend could be observed between childhood adversity and the total fixation duration for angry (*r* = − 0.36, *p* = 0.08), and for fearful faces (*r* = − 0.38, *p* = 0.06). For carriers of the MAOA-H polymorphism no significant relations between childhood adversity and emotional facial expressions were found (all *p*s > 0.2).

### Temperament, child behaviour, childhood adversity and attention biases

Externalizing/internalizing behaviour problem scores were associated with childhood adversity (*r*_extern_ = 0.47, *p* < 0.01; *r*_intern_ = 0.29, *p* = 0.03) while no relationship between externalizing/internalizing behaviour problems and attention bias for each emotional factor score (negative emotional valence, positive emotional valence) was observed (in the total sample and in each genetic group, all *p*s > 0.2).

## Discussion

Spontaneous and observable attention biases were assessed to explore underlying neurocognitive mechanisms of childhood adversity. Childhood adversity interacted with the MAOA genotype, indicating an attention bias away from negative (especially sad) stimuli with increasing childhood adversity levels. Apart from this avoidance-related effect, boys with increasing childhood adversity levels generally preferred to look at positive emotional stimuli (approach-related effect). Neither age nor children’s internalizing/externalizing behaviour had a substantial influence on these main results.

Previous studies on attention biases associated with childhood adversity reported mixed results. For example, in children with early maltreatment experience, an attention bias away from negative facial expressions was observed (Pine et al. 2005), in particular from sad faces (Mastorakos and Scott 2019). In contrast, another study reported an attention bias towards sad facial stimuli in maltreated children when sad mood was induced (Romens and Pollak 2012). In this case, heterogeneous findings related to the direction of the attention bias could be explained by the specific mood induction procedure. Indeed, adolescent negativity has recently been proven as a latent factor, which induces an attention bias towards negative faces (Harrewijn et al. 2021), supporting the argument given above.

In addition, genetic moderators might modify the severity of distress sensations leading to opposite gaze directions (Owens et al. 2016). For example, the direction of the attention bias in children suffering from maternal criticism (as a chronic stressor) was found to be moderated by 5-HTTLPR. Children carrying at least one short allele avoided angry faces if maternal criticism was high while children homozygous for the long allele demonstrated an attention bias towards angry faces (Gibb et al. 2011). The reported attention bias away from angry faces was explained by stronger amygdala reactivity to facial expressions of emotion (Munafò et al. 2008) and with greater stress sensitivity (Gotlib et al. 2008) due to the serotonin transporter gene.

In a similar vein, the influence of the MAOA-genotype on amygdala responsivity was investigated while healthy participants were viewing threat-related (angry and fearful) faces. An enhanced amygdala reactivity was demonstrated in male carriers of the MAOA-L polymorphism in contrast to the MAOA-H polymorphism (Meyer-Lindenberg et al. 2006), which was replicated for increasing childhood adversity levels in male MAOA-L carriers (Holz et al. 2016).

Interestingly, an avoidance tendency was repeatedly reported in children (Boyd et al. 2006; Kujawa et al. 2011) who were parented by a depressive mother, which might represent a strategy for emotion regulation in young children. As unhappiness and chronic distress are explicitly assessed in the CAPI, it may be that some parents frequently display a sad mood and children try to avoid sadness by looking away (especially from sad facial expressions).

An attention bias towards positive stimuli has previously been reported in one study investigating maltreatment severity and attachment anxiety. In this study, participants with a severe abuse history and low attachment anxiety paid more attention to happy faces (Davis et al. 2014). Other studies failed to find any relationship between positive stimuli and a history of maltreatment (Pine et al. 2005), while a protective bias towards positive stimuli is well known in remitted depressive adults (Li et al. 2016) and in healthy adults when sad mood was induced (Isaacowitz et al. 2008).

Although little research exists on the relationship between childhood adversity and the attention bias towards positive emotional stimuli, it has been reported that children with early neglect exhibit decreased positive affect (Stellern et al. 2014). Moreover, dysfunctional neural mechanisms of reward processing were observed in adults with adverse childhood experiences (Dillon et al. 2009; Mehta et al. 2010). Therefore, it might be that children suffering from chronic distress regulate and enhance their mood by exhibiting an attention bias towards positive emotional stimuli (Harrison and Gibb 2015).

The genotype groups in this study differed with respect to externalizing and internalizing problem behaviour. Notwithstanding, a relationship between childhood adversity and externalizing/internalizing problem behaviour was observed, which is known to represent a bidirectional association. Accordingly, increased externalizing/internalizing problem behaviour in the MAOA-H group is most probably attributable to the tendency towards higher childhood adversity levels (or vice versa). On the opposite side, childhood adversity might lead to attention biases away from negative facial expressions and towards positive facial expressions (while the reversed association is implausible). Hence, we argue that boys exhibit an avoidance-related behaviour, which is slightly enhanced in the MAOA-L group, even though the MAOA-H group exhibited more externalizing/internalizing problem behaviour. Consequently, the most probable explanation for the missing relation between attention biases and child behaviour (CBCL)/children’s temperament (JTCI) is that children were not in an (experimentally induced) angry or sad state in the present study (Bodenschatz et al. 2018).

In sum, the approach-related behaviour and the avoidance-related behaviour suggest emotional regulation capabilities in children. However, a failure of this emotion regulation strategy (e.g., as we hinder children) might yield anger accompanied by impulsive-aggressive behaviour and decreased mood (Nozadi et al. 2018). Therefore, the so-called hostile attention bias (or the negative attention bias) might be a consequence of anger (and decreased mood) which eventually serves for the maintenance of an aggressive (or depressive) behavioural style (Nozadi et al. 2018).

## Limitations and conclusions

A major limitation of this study is the small sample size. Especially in genetic studies small effect sizes are usually expected, which typically calls for large-scale studies. Here, it might be that the MAOA-L genotype only slightly enhanced the childhood adversity effect leading to an environment x genotype interaction. Moreover, it is possible that the present eye-tracking approach is more sensitive than typical behavioural designs. Regardless of these arguments, replication studies are highly recommended.

Another limitation is that in some portion the observed effect might be related to altered face recognition processes in children suffering from childhood adversity experiences (e.g. Pollak and Sinha 2002; Pollak et al. 2000). As a neutral and an emotional facial expression was presented simultaneously in this study, children might have difficulties to distinguish neutral from emotional facial expressions with increasing childhood adversity levels. Although modified face recognition processes are conceivable, a recent large-scale study investigating facial recognition failed to find any effects of childhood adversity in children (Dunn et al. 2018.)

One distinctive strength of our study is the eye-tracking approach, which enabled us to investigate attention biases as spontaneous behavioural measures, which become directly observable even in small sample sizes. The avoidance and approach-related effect could serve as an intermediate phenotype especially for children, which might provide insight into underlying neurocognitive mechanisms related to the avoidance and the approach system. Moreover, with respect to the attention bias away from negative faces, our results might not be explained by the children’s difficult temperaments or behaviour, as children scored higher on externalizing and internalizing behaviour in the genetic non-risk group (MAOA-H).

In conclusion, an attention bias away from negative emotional stimuli was observed in boys carrying the MAOA-L polymorphism, indicating a stronger avoidance effect with increasing childhood adversity levels especially for sad faces. In contrast, we detected a more general approach tendency towards positive emotional facial expressions in children who were increasingly exposed to adverse environmental circumstances. While the avoidance-related effect can likely be explained within the framework of heightened stress sensitivity, the approach effect might be associated with a modulated reward system and decreased positive affect. Both tendencies—the approach-related behaviour and the avoidance-related behaviour—suggest emotional regulation capabilities in children experiencing adverse circumstances (e.g. Mastorakos and Scott 2019).

## Data Availability

Data will be disclosed on request and after approval of the proposed use of the data by the study committee.
